# Do Mast Cells Have a Role in Tendon Healing and Inflammation?

**DOI:** 10.3390/cells9051134

**Published:** 2020-05-04

**Authors:** Md Abdul Alim, Magnus Peterson, Gunnar Pejler

**Affiliations:** 1 Department of Public Health and Caring Sciences, General Medicine, Uppsala University, 751 22 Uppsala, Sweden; Magnus.Peterson@pubcare.uu.se; 2 Department of Medical Biochemistry and Microbiology, Uppsala University, 75123 Uppsala, Sweden; 3 Academic Primary Health Care, Region Uppsala, Sweden; 4 Department of Anatomy, Physiology and Biochemistry, Swedish University of Agricultural Sciences, 756 51 Uppsala, Sweden

**Keywords:** tendon healing, mast cells, neuropeptides, inflammation, tendon pain

## Abstract

Understanding the links between the tendon healing process, inflammatory mechanisms, and tendon homeostasis/pain after tissue damage is crucial in developing novel therapeutics for human tendon disorders. The inflammatory mechanisms that are operative in response to tendon injury are not fully understood, but it has been suggested that inflammation occurring in response to nerve signaling, i.e., neurogenic inflammation, has a pathogenic role. The mechanisms driving such neurogenic inflammation are presently not clear. However, it has recently been demonstrated that mast cells present within the injured tendon can express glutamate receptors, raising the possibility that mast cells may be sensitive to glutamate signaling and thereby modulate neurogenic inflammation following tissue injury. In this review, we discuss the role of mast cells in the communication with peripheral nerves, and their emerging role in tendon healing and inflammation after injury.

## 1. Introduction

Mast cells are found as resident cells in most tissues of the body, with a particular abundance at sites close to the exterior, such as skin and mucosal surfaces. This is in line with a proposed role of mast cells as sentinel cells capable of responding rapidly to external insults such as bacterial and viral infection, and in the host defense against various toxins [1,2]. In addition, mast cells are found in supporting tissues such as tendon [3,4,5] and bone [6,7], and also in skeletal muscle [5,8,9]. However, their role in these tissues is not fully understood. Notably, mast cells are frequently found in the vicinity of peripheral nerve endings. Based on this, it has been proposed that mast cells can have a role in nerve signaling, for example, by secreting mediators that can activate nearby nerves and thereby initiate signaling events that are transmitted to the central nerve system. Potentially, such mast cell:nerve communication could have a role in mediating various responses in supporting tissues, including signaling leading to pain perception in the context of tendon pathology. Regarding the presumed mechanisms by which mast cells communicate with nerve endings, several scenarios have been suggested. One of these, based on recent evidence, is that signaling through glutamate could be one potential mechanism operative in injured tendon [5,10]. Based on this recent development, we discuss the emerging role of mast cells in tendon healing and inflammation following injury.

## 2. Tendon

Tendon is a crucial component of the musculoskeletal system that connects muscle to bone and transmits force for the movement [11,12]. Tendon is a soft connective tissue and is predominately composed of water, which makes up 55–70% of the total tendon weight. The other major component of tendon is collagen, which represents about 60–85% of the dry weight of tendon [13]. In tendon architecture, fibrillar arrangement of triple-helical type I collagen molecules generates collagen fibers, which then combine to form fascicles and, ultimately, the tendon tissue. The type I collagen molecule contains two identical α1 chains and one α2 chain, which are encoded by *col1a1* and *col1a2,* respectively [12]. The collagen fibrils are the fundamental force-transmitting element of tendon tissue, and are tightly arranged within the extracellular matrix. Type I collagen and associated extracellular matrix components are produced by tenocytes, which are fibroblast-like cells found between collagen fibers and in the surrounding of the endotenon [14]. In addition to collagen, other molecules like elastin and proteoglycans are also integral parts of the tendon [15]. There are two main markers of collagen metabolism: procollagen type III N-terminal propeptide (PIIINP) and procollagen type I N-terminal propeptide (PINP). Both have been used as early prediction markers for healing tendon and bone [16]. Procollagen type I and III are essential building blocks in all types of connective tissues, and PINP and PIIINP have been utilized as biomarkers to assess collagen metabolism in intact human Achilles tendons exposed to exercise and growth factor stimulation [16]. 

## 3. Tendon Healing and Inflammation 

Tendon injury and tendon pain can develop gradually over time or rapidly with overuse or overload, the latter being exemplified by Achilles tendon injury. The inflammatory reaction to acute injury, traditionally called tendinitis, has been challenged by the notion that chronic tendon pain has a different histologic appearance, for which a different term of tendinosis has been suggested [17,18,19,20]. For practical clinical purposes, when histologic examination is not possible, both tendinitis and tendinosis are often grouped together and termed tendinopathy [18,20]. Other common examples of tendon injury and tendon pain are tennis elbow (lateral epicondylitits), golfers elbow (medial epicondylitis), and jumper’s knee (patellar tendinitis). Typically, tendon injury can occur as a result of gradual wear and tear and/or by repetitive motions, and is accompanying the ageing processes of the tendon [12,21,22]. Tendon injury and tendon pain is also affected by underlying diseases such as arthritis, infections, diabetes, and thyroid disease [23,24]. The exact mechanisms behind the inflammatory response following tendon injury are not fully understood. However, emerging evidence suggests that alarmins released from necrotic cells can constitute important triggers for the ensuing inflammatory response [25]. As part of ongoing inflammation and delayed healing, the histologic appearance will contain irregularities from healthy tendon tissue such as calcifications and sprouting of nerves and vessels along with the upregulation of signaling peptides and receptors such as substance P, calcitonin gene-related peptide (CGRP), and N-methyl-D-aspartate (NMDA) receptors [5,10,26,27,28,29,30,31,32].

**Figure 1:**
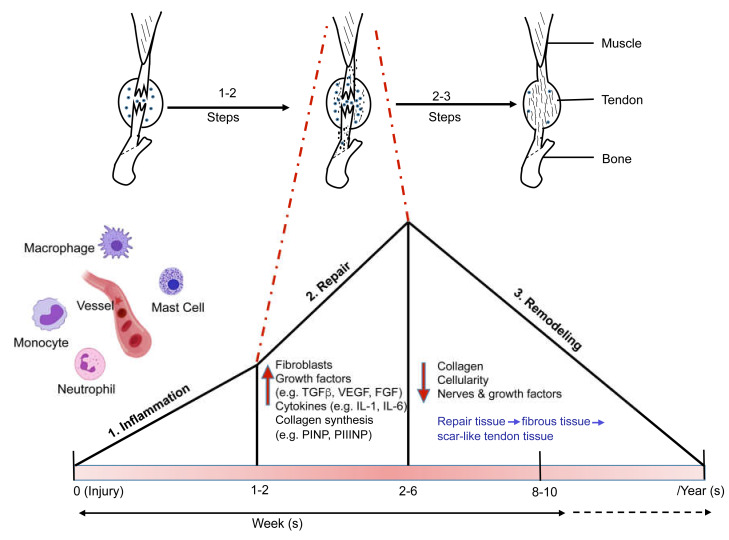
Phases of tendon healing: inflammation, repair, and remodeling. During the inflammatory phase, immune cells (macrophages, neutrophils, and mast cells) predominate. The inflammatory phase is followed by the proliferation or repair phase where fibroblasts produce collagens and extracellular matrix components. The proliferation phase is followed by a remodeling phase, in which the tendon modifies its internal structure.

After injury, the tissue repair process normally follows three overlapping phases, described as the (i) inflammatory, (ii) proliferative, and (iii) remodeling phases (Figure 1) [24,33,34]. During the inflammatory phase (within 2 weeks from injury), immune cells including macrophages, neutrophils, and mast cells predominate. In this phase, different vasoactive factors and cytokines drive the inflammation by promoting vascular leakage and migration of leucocytes, primarily neutrophils, to the inflammatory site [35,36,37]. In most cases, the inflammatory processes are gradually resolved, but can in some cases proceed to a chronic inflammatory stage. Such chronic tendon inflammation is considered as a protracted, dysregulated, and maladaptive response to injury. The inflammatory phase is followed by a proliferative, or repair, phase (2–6 weeks). In this phase, fibroblasts produce collagens (e.g., procollagen type I, PINP; and procollagen type III, PIIINP) and other extracellular matrix components [38]. The proliferation phase is followed by a remodeling or maturation process (6 weeks–2 years), in which the tendon structure is modified [39]. The molecular events during these healing phases are influenced by different factors such as site of injury, age, sex, genetics, and nutrition [34,40,41].

The pathological mechanisms of tendon healing are still far from understood. In peripheral tissues, the peripheral nerve system has a key role in regulating inflammation and pain signaling of the damaged tissue via afferent to efferent pathways [42]. In the healthy tendon, nerve fibers are localized in the tendon sheath, so called interfibrillar matrix, whereas the tendon proper, also denoted the intrafibrillar matrix, is devoid of nerves [26]. During the early healing phase, extensive ingrowth of nerve fibers has been demonstrated into the tendon proper [43]. During the repair phase there is also nerve ingrowth in the tendon proper, but in the subsequent phase (remodeling phase) the nerves retract back to the surrounding tendon sheath (Figure 1) [28]. 

## 4. Mast Cells

**Figure 2:**
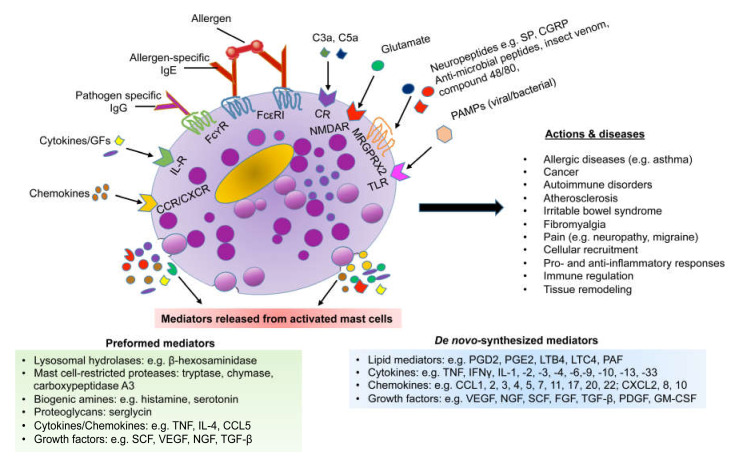
Mast cells are activated by multiple stimuli and secrete both preformed and de novo-synthesized mediators in response to activation. SP, substance P; CGRP, calcitonin gene-related peptide; PAMP, pathogen-associated molecular pattern; GF, growth factors, CR, complement receptor; MRGPRX2, Mas-related G-protein coupled receptor member X2; NMDAR, N-methyl-D-aspartate receptor; PG, prostaglandin; LT, leukotriene; VEGF, vascular endothelial growth factor; NGF, nerve growth factor; SCF, stem cell factor; FGF, fibroblast growth factor; PDGF, platelet-derived growth factor.

Mast cells are highly granulated hematopoietic cells derived from the bone marrow. They circulate in the blood as immature progenitor cells, after which they home into tissues where they mature under the influence of local growth factors such as stem cell factor [44,45,46,47]. In their mature state, mast cells are characterized by a remarkably high content of electron-dense secretory granules. These contain a plethora of preformed mediators, including serglycin proteoglycans, proteases (e.g., chymase, tryptase, and carboxypeptidase A3), biogenic amines (histamine, serotonin, and dopamine), lysosomal hydrolases, growth factors, and certain cytokines (e.g., tumor necrosis factor-α (TNF-α)) [48,49,50]. When mast cells are activated, they typically respond by degranulation, whereby the preformed mediators are released to the extracellular space. Mast cell activation also leads to the de novo synthesis of a range of other mediators, including additional growth factors, cytokines, chemokines, as well as various lipid-derived mediators such as platelet-activating factor, prostaglandins, and leukotrienes (see Figure 2) [50]. Mast cells can be activated through a variety of mechanisms. Of these, crosslinking of IgE molecules bound to their high affinity receptors (FcεRI) on the mast cell surface represents the classical mode of mast cell activation. However, mast cells can be activated through several alternative pathways, including complement, engagement of toll-like receptors, and ligation of Mas-related G-protein coupled receptor member X2 (MRGPRX2) [2,51,52,53,54]. 

Mast cells have been suggested to have a number of beneficial functions, e.g., in the context of bacterial and parasite infection, as well as in wound healing and in the defense against various toxins [55,56,57,58,59]. Conversely, mast cells have also been implicated as detrimental players in several pathological settings. Most notably, mast cells are strongly implicated in allergic disorders but there is also evidence supporting the contribution of mast cells in various autoimmune diseases, fibrosis, cancer, skin inflammation, and metabolic disorders [48,60,61,62,63,64,65,66,67]. In addition, there is emerging evidence suggesting that mast cells potentially could be involved in conditions associated with neurogenic inflammation [51,68,69,70,71]. In support of a functional nerve:mast cell communication, mast cells are frequently found in close association with nerve endings [69,70,71]. However, the mechanisms by which mast cells could respond to nerve signaling have been mostly elusive.

## 5. Mast Cells in Tendinopathy 

Mast cells are in the normal state resident locally in the tendon tissue or in the loose connective tissue close to the paratenon, muscle–tendon junction, or bone–tendon junction [5]. However, during the tendon healing process, they may migrate to the injured site (tendon proper) following inflammation and nerve ingrowth [5,72]. At present it is not clear whether the increase in mast cells numbers in the injured tendon is solely a result of mast cell migration from distant sites, or if the injury is accompanied by mast cell proliferation or influx of mast cell progenitors from the circulation. 

The precise role of mast cells in tendinopathy is intriguing. As noted above, activated mast cells have the capacity to secrete a wide panel of bioactive compounds, both from preformed stores and following de novo synthesis [48,50]. Notably, several of these released compounds could potentially influence the inflammatory and proliferative healing phases after tendon injury. For example, mast cell-derived vascular-endothelial growth factor (VEGF) and nerve growth factor (NGF) may contribute to neo-angiogenesis and nerve ingrowth [73,74]. Of particular relevance considering that tendon is predominantly composed of collagen, there is large documentation suggesting a role for mast cells in the regulation of collagen turnover. Firstly, mast cells are known to produce growth factors, including TGFβ and FGF2, which stimulate collagen synthesis in fibroblasts [75,76,77]. Potentially, such mast cell-derived growth factors could also stimulate collagen synthesis in tenocytes, but this remains to be demonstrated. It is also known that proteases released from mast cells, in particular tryptase and chymase, can stimulate collagen synthesis in fibroblasts [78,79,80,81,82], and it is thus reasonable to assume that these proteases can have similar impact on tenocytes. In addition to promoting collagen synthesis, mast cells can also contribute importantly to the degradation of collagen. This is primarily manifested by the ability of mast cell-expressed proteases to activate various members of the matrix metalloprotease family, including procollagenases [83,84,85,86]. Altogether, mast cells thus have the capacity to both promote and dampen collagen deposition, suggesting a complex regulatory impact on connective tissue remodeling. However, it remains to be investigated if such mast cell-dependent effects on collagen turnover are operative in the context tendon inflammation/healing. 

The close location of mast cells and peripheral nerve endings raises the possibility that mast cells can be activated by different neurotransmitters that may be released from peripheral nerves in response to tendon injury. Such neurotransmitters include substance P, glutamate, CGRP, and neurokinin A (NKA) [26,87,88]. In line with a potential role for neurological mechanisms in mast cell activation, mast cells are known to express several receptors for neurotransmitters, e.g., neurokinin 1 receptor (NK1; receptor for substance P) and calcitonin receptor-like receptor (receptor for CGRP) [89,90,91]. Mast cells also express MRGPRX2 [54], and it has been shown that MRGPRX2 can be a more relevant receptor for substance P than is NK1 [92]. Mast cells also express corticotropin-releasing hormone receptor-1 and activity-modifying protein 1 (RAMP1) [93,94]. Further, recent findings have revealed that mouse mast cells express various glutamate receptors [10] (see also below). Altogether, this suggests that mast cells have the capacity to respond to a wide range of neurotransmitters that can be secreted by nerve endings in the context of tendon injury. Such neurotransmitters could potentially activate mast cells, and could also activate other cells (e.g., macrophages, fibroblasts) expressing the corresponding neurotransmitter receptors. This can lead to the release of cytokines and other proinflammatory mediators that could contribute to the pathology of tendon injury [95,96,97,98]. Indeed, mast cells are known to respond to substance P stimulation by secreting monocyte chemoattractant protein-1 (MCP-1), TNF-ɑ, interleukin-8 (IL-8), IL-3, granulocyte–macrophage colony-stimulating factor, interferon-γ, and eotaxin [99,100]. Moreover, mast cell stimulation by CGRP and substance P causes the release of histamine from rat peritoneal mast cells [101] but does not activate human intestinal mast cells [102]. It has also been demonstrated that mast cells respond to glutamate by secreting proinflammatory cytokines and chemokines [10].

In addition to acting as potential sensors for neurotransmitters secreted from nerve endings, mast cells have also the capacity to act in the opposite direction, i.e., to activate peripheral nerve cells. This can be accomplished by the secretion of various neurotransmitters—histamine, serotonin, and dopamine, which can activate the cognate receptors expressed by neurons. In line with this scenario, upregulation of histamine receptor 1 in injured afferents may be involved in neuropathic pain [103,104]. Moreover, serotonin (5-HT3_A_) and dopamine (D1-like and D2-like) receptors are also expressed by peripheral nerve endings [105,106], and may thus be engaged by the corresponding ligands released by activated mast cells.

Another potential scenario is that mast cell-expressed proteases could act on nerves. In particular, it has been suggested that mast cell tryptase can influence peripheral nerves by activating protease-activated receptor 2 (PAR-2), expressed on the surface of neurons [107,108]. Notably, tryptase expression is essentially restricted to mast cells [109,110], and the activation of PAR-2 by tryptase is thereby a mast cell-dependent process. From a different angle, there is the possibility that mast cell-expressed proteases could have a down-regulating impact on signaling from nerves, by degrading various neurotransmitters. The latter is exemplified by the ability of mast cell chymase to degrade substance P [111] and by the capacity of tryptase to degrade CGRP [112]. Hence, the mast cell proteases could potentially have a complex regulatory role in nerve signaling, being able both to trigger and dampen activation of nerve signaling.

**Figure 3:**
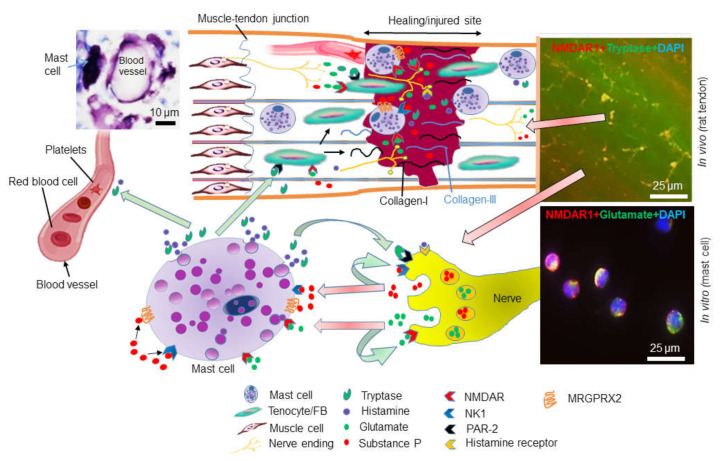
A possible role of mast cells in regulating the inflammatory and healing responses in the injured tendon. After tendon injury, peripheral nerve endings can release neuropeptides, e.g., glutamate and substance P, that may activate mast cells or fibroblast-like cells (tenocytes) via their respective receptors, e.g., glutamate receptors (e.g., NMDAR1) NK1 and MRGPRX2. Activated tenocytes can proliferate and increase their type-I and type-III collagen synthesis during tendon healing. Activated mast cells can release proteases, e.g., tryptase, which may have a functional impact on tendon cells or may activate nearby nerves via PAR-2. Mast cells can also activate nerves via their release of histamine, which can bind to histamine receptors on nerve endings. Further, mast cells can also affect angiogenic processes. The upper right panel shows the colocalization of NMDAR1 with tryptase in vivo in injured tendon; the lower right panel shows colocalization of glutamate and NDMAR1 in glutamate-stimulated mast cells. The upper left image depicts the close localization of mast cells to blood vessels in injured tendon.

Altogether, mast cells thus have the capacity both to activate neurons and to respond to transmitters secreted by nerve endings. To add complexity, there is the possibility that the nerve cell:mast cell axis could generate an amplifying loop. Such an amplifying loop could be initiated through activation of mast cells, causing the release of mediators that activate nerve cells. The activated nerve cells then would respond by secreting transmitters that could cause an enhanced activation state of the mast cells, in turn causing elevated release of nerve-activating mast cell mediators (Figure 3). Alternatively, the process could be initiated by the release of mast cell-activating neurotransmitters from nerve endings. This could potentially lead to a chronic state with continuous activation of both nerve cells and mast cells, potentially contributing to the features of chronic tendon injury, such as sustained inflammation and pain. We may thereby envision that strategies aimed to interfere with mast cell activation in the injured tendon could potentially be exploited for therapeutic purposes. For example, it could be possible to dampen tendon inflammation by administration of various mast cell stabilizers, i.e., compounds that inhibit mast cell degranulation. Further, inhibition of the mast cell proteases could represent another potential therapeutic strategy. Antihistamines could also be exploited for such a purpose [113].

A role of mast cells in tendon healing processes is also supported by human clinical studies. In one study, it was shown that the numbers of mast cells increased approximately three-fold in tendinopathic tissue from patients suffering from patellar tendinosis vs. corresponding tissue from control patients undergoing intramedullary nailing of the tibia [3]. Notably, mast cells were more frequent in the injured tissue than were either macrophages or lymphocytes, suggesting that mast cells represent a dominating inflammatory cell population of the injured tendon. Further, it was shown that mast cells were predominant in the tendon proper and that they were closely associated with neovessels [3], the latter arguing that mast cells may have an influence on the vessel ingrowth in the inflamed tendon. It was also observed that mast cell density was correlated with the vessel-area fraction, and that particularly high mast cell numbers were seen in patients with a long reported symptom duration [3]. In another study, it was shown that the numbers of mast cells were increased in samples from torn tendon samples vs. controls. Here, an approximately two-fold increase in mast cell numbers was seen in injured vs. control tissue [114]. In agreement with the study from Scott et al. [3], mast cells were predominantly located close to vessels, and a link between mast cells and angiogenesis was suggested [114]. An increase in mast cell density in the context of tendinopathy is also supported by a study where the cellular and vascular changes in different stages of full thickness tears of the rotator cuff were followed [115]. 

In further support for a role of mast cells in tendon pathology, an increased number of mast cells has been observed in rabbits after deep flexor tendon repair [116], and in the tendinopathy seen in the calcaneal tendon overuse rat model [4]. Moreover, it was shown in a recent study that increased numbers of mast cells were found in the injured rat Achilles tendon 3 weeks post injury [5]. A major finding of the latter investigation was that mast cells of the injured tendon showed signs of activation, as evidenced by extensive degranulation. Moreover, mast cell activation was prominent in all investigated areas of the healing tendon, i.e., the muscle–tendon junction, mid-tendon, and bone–tendon junction, suggesting that tendon healing is associated with widespread activation of mast cells [5].

## 6. Glutamate Receptors in Tendinopathy and Mast Cells

Glutamate is the primary excitatory mediator of the nervous system as well as of non-neuronal cells [117]. It acts by binding to various glutamate receptors, subdivided into metabotropic and ionotropic types. The metabotropic glutamate receptors are G protein-linked and include the group I (mGlu1R and mGlu5R; coupled to Gq/G11 proteins), group II (mGlu2 and mGlu3; coupled to Gi/Go proteins), and Group III (mGlu4, mGlu6, mGlu7 and mGlu8 receptors; coupled to Gi/Go proteins) metabotropic receptors [118,119,120]. The ionotropic glutamate receptors are ion channel-linked and are grouped into four distinct classes based on pharmacology and structural homology: the NMDA receptors (NMDAR1, NMDAR2A-NMDAR2D, NMDAR3A, NMDAR3B) [121,122,123,124], the AMPA receptors (GluA1-GluA4) [125,126], the kainate receptors (GluK1-5) [127,128], and the δ receptors (GluD1 and GluD2) [122].

Glutamate:glutamate receptor signaling has been implicated in various pain conditions, including tendinopathy [17,30,129]. For example, the glutamate receptor NMDAR1 has been identified in tendinopathy. This was shown in a study of tendinopathic patients, where a 10-fold up-regulation of NMDAR1 was observed in morphologically transformed tenocytes, in the endothelial and adventitial layers of neovessel walls and in presumed sprouting nerve fibers [130]. It is now recognized that nerve ingrowth into the tendon, and the upregulation of glutamate receptors such as NMDAR1, is part of both the physiological and pathological consequences of tendon injury but also a likely mechanism underlying chronic tendinopathy, such as pain.

Intriguingly, there is evidence to suggest that glutamate receptors can be expressed not only by neurons but also by diverse inflammatory cells that can populate the injured tendon. For example, it has been demonstrated that rodent lymphocytes and microglia express mGlu receptors in response to glutamate [131,132]. Mechanistically, stimulation of glutamate group I metabotropic glutamate receptors was shown to induce calcium signaling and c-fos gene expression in human T cells [133]. It has also been demonstrated that activation of mGlu2 receptors in microglia causes upregulated expression of proinflammatory cytokines, hence mediating neurogenic inflammation [134]. 

In a recent study, we shed further light on this issue by studying the possible impact of glutamate receptor expression in mast cells and the possible function this could have [5,10]. In this study, we demonstrated that exposure of mouse mast cells to glutamate activates a transient expression of a range of glutamate receptors, of both ionotropic and metabotropic type [10]. There is also limited evidence from earlier studies that mouse mast cells may respond to glutamate receptor antagonists [135]. Further, we observed that the exposure of mast cells to glutamate led to an upregulated expression of proinflammatory cytokines/chemokines, introducing the concept of a glutamate:glutamate receptor axis in mast cells that can contribute to neurogenic inflammation. We also noted that glutamate stimulation of mast cells resulted in upregulated expression of a number of transcription factors, in particular FosB [10], with implications for various processes, including inflammation and tissue homeostasis [136]. In line with an impact of FosB on tendon pathology, FosB expression has been shown to be mechanosensitive at both the mRNA and protein level, in various cell types including tenocytes [137,138,139]. It is also interesting to note that FosB can be upregulated by different kinds of stimuli in rodent and human cells, including wounding [140,141]. Altogether, FosB is now emerging as a major upregulated gene in the context of tendon injury, and our recent findings suggest that mast cells could represent major FosB-expressing cells in such settings. 

Overall, glutamate signaling emerges as a potential pathogenic mechanism operative in tendon injury, and strategies to target glutamate receptors could thereby represent potential therapeutic options in tendinopathy or other conditions with an involvement of neurogenic inflammation [142].

## 7. Concluding Remarks

As discussed in this review, there is emerging evidence to suggest that mast cells can contribute to neurogenic inflammation and the inflammatory reaction that accompanies tendon healing. It is plausible that the cytokines/chemokines released through this mechanism may contribute, either directly or indirectly, to the modulation of tendon healing and inflammation in such settings. Further, it is possible that a glutamate:glutamate receptor axis can account for mast cell activation in the context of tendon injury. However, further studies are required to fully establish the emerging role of mast cells in mediating physiological and pathophysiological responses in tendon healing after injury, and to evaluate whether the glutamate:glutamate receptor axis in mast cells can be exploited for therapeutic purposes in tendinopathy and tissue healing.
